# Cognitive Modeling of Individual Variation in Reference Production and Comprehension

**DOI:** 10.3389/fpsyg.2016.00506

**Published:** 2016-04-07

**Authors:** Petra Hendriks

**Affiliations:** Center for Language and Cognition Groningen (CLCG), University of GroningenGroningen, Netherlands

**Keywords:** ACT-R, cognitive modeling, perspective taking, processing speed, reference comprehension, reference production, working memory

## Abstract

A challenge for most theoretical and computational accounts of linguistic reference is the observation that language users vary considerably in their referential choices. Part of the variation observed among and within language users and across tasks may be explained from variation in the cognitive resources available to speakers and listeners. This paper presents a computational model of reference production and comprehension developed within the cognitive architecture ACT-R. Through simulations with this ACT-R model, it is investigated how cognitive constraints interact with linguistic constraints and features of the linguistic discourse in speakers’ production and listeners’ comprehension of referring expressions in specific tasks, and how this interaction may give rise to variation in referential choice. The ACT-R model of reference explains and predicts variation among language users in their referential choices as a result of individual and task-related differences in processing speed and working memory capacity. Because of limitations in their cognitive capacities, speakers sometimes underspecify or overspecify their referring expressions, and listeners sometimes choose incorrect referents or are overly liberal in their interpretation of referring expressions.

## Linguistic Reference

An important function of language is reference. Speakers refer to things, people, or events in the world around them and listeners identify these referents based on the referring expressions used by the speaker. To refer, speakers can use a variety of forms. For example, to refer to their neighbor they could utter the indefinite noun phrase *a lady who lives next door* or the definite noun phrase *the lady who lives next door*, refer to her by her name, use a personal pronoun such as *she* or *her* or a reflexive pronoun such as *herself*. Which form a speaker decides to use depends on a large number of factors, including the structure of the sentence and the prominence of the referent in the context of utterance. Likewise, to interpret the referring expression uttered by the speaker, listeners can often choose between various referents. This choice also depends on various factors.

Reference has been a central topic in many subfields of linguistics over the past decades. Although the factors influencing speakers’ production and listeners’ comprehension of referring expressions have been studied extensively from various angles, there does not exist a comprehensive account of linguistic reference yet. One of the main challenges for such a comprehensive account is the observation that speakers vary considerably in their choice of referring expression. This variation is problematic for most theoretical and computational models of linguistic reference. Theoretical models of referential choice (e.g., Gundel et al., 1993, 2012) generally attribute this variation to the interaction of the model with general cognitive and pragmatic factors and principles, without offering a specification of how the variation arises. Most computational algorithms for the generation of referring expressions are deterministic (van Deemter et al., 2012) and therefore always generate the same referring expression in a particular situation (but see Frank and Goodman, 2012; van Gompel et al., 2012; Mitchell et al., 2013, for recent probabilistic approaches). As a consequence, no variation is produced. Even if different individual speakers are modeled by different computational algorithms (as proposed by Dale and Viethen, 2010), variation within speakers is not accounted for.

This paper addresses the question of how the observed variation in speakers’ choice of referring expression and listeners’ choice of referent (which is discussed below) can be accounted for. It is hypothesized that at least part of this variation can be explained as resulting from the dynamic interaction between linguistic and cognitive constraints on reference. The application of these constraints is dependent on the cognitive capacities of speakers and listeners. Cognitive capacities can vary between individuals (e.g., between children and adults), but also within the same individual (e.g., due to linguistic and cognitive development or as an effect of the task). For example, some tasks are cognitively more demanding than other tasks and will therefore leave the speaker or listener with insufficient cognitive resources to make an optimal referential choice. A promising approach to investigate this hypothesis is by computational cognitive modeling of linguistic reference. Using computational cognitive modeling, models are developed that are cognitively plausible rather than computationally optimal. Hence, they incorporate the normal variability found in human performance.

In the next section, we discuss different types of variation that have been observed in the psycholinguistic literature on referential choice. Next, the cognitive architecture ACT-R is introduced. Within this cognitive architecture, a computational cognitive model of reference has been developed. It is shown how this ACT-R model is able to account for speakers’ underspecification and overspecification of referring expressions and listeners’ incorrect interpretations of referring expressions in particular tasks.

## Variation in Referential Choice

Psycholinguistic investigations of the referential choices made by human speakers reveal considerable variation, both among and within individuals and across tasks. For example, in a web-based experiment where adult participants were asked to produce referring expressions to describe one of three objects shown in a picture in such a way that a friend looking at the same picture would be able to identify the target referent, speakers were found to show a large amount of variation in the referring expressions they produced (Dale and Viethen, 2010). For the same visual scene, different speakers produced different forms, such as *the blue cube*, *the blue cube in front of the red ball*, and *the cube in front of the ball*. Many of these forms were overspecific and informationally redundant. A few were not specific enough and failed to uniquely distinguish the target referent from the other two objects. This illustrates that, even in the very same task, speakers overspecify as well as underspecify their referring expressions. Similar patterns of frequent overspecification and some underspecification of referring expressions were found in other psycholinguistic studies (e.g., Deutsch and Pechmann, 1982; Engelhardt et al., 2006; Koolen et al., 2011).

In addition to variation among speakers, it has been observed that there is also variation within speakers. van Deemter et al. (2012, p. 174) examined the data of Fukumura and van Gompel (2010), who carried out two written sentence completion experiments with adult participants to investigate the choice between a pronoun and a name for a previously mentioned referent. Van Deemter and colleagues found that the majority of participants in the study of Fukumura and van Gompel did not produce only pronouns or only names, but produced both types of referring expression in at least one of the conditions of the experiments.

**FIGURE 1:**
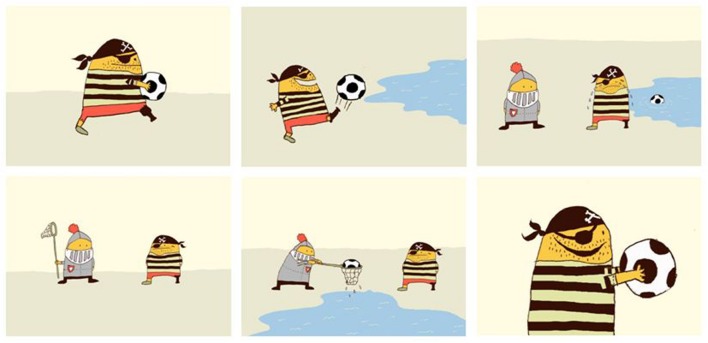
**Picture story used for eliciting narratives in the studies of Hendriks et al. (2014) and Kuijper et al. (2015)**.

Variation in speakers’ referential choices can also be observed in more naturalistic tasks, such as telling a story. Reference production during story telling is often investigated on the basis of cartoon movies or picture books (e.g., Karmiloff-Smith, 1985; Arnold et al., 2009). For example, picture stories like **Figure 1** were used to elicit narratives in children, young adults and elderly adults (Hendriks et al., 2014), and in children with autism, children with ADHD and typically developing children (Kuijper et al., 2015). The picture stories in these experiments featured two characters of the same gender and were designed to elicit two topic shifts: one halfway through the story (when the second character enters the story) and the other one at the end of the story (when there is a shift in focus to the first character again). The participants were instructed to tell the story so that a second experimenter, who could not see the pictures, would be able to understand the story.

On the basis of the picture story in **Figure 1**, participants produced narratives such as the following (from Hendriks et al., 2014, translated from Dutch and slightly adapted for the sake of readability):

Speaker 1: A pirate with the football. Then he kicks it. Then it is in the water. Then the knight goes to catch it. And he has caught the ball in a net. Now he has his ball back again.
Speaker 2: The pirate with a wooden leg has a football. He kicks the football with his wooden leg into the pond. And cries because he can’t reach the ball anymore. The knight sees all that. The knight gets a net. And gets the ball out of the water for the pirate. The pirate has a big smile because he is happy that he has the ball back again.

The narrative produced by speaker 2 differs from the narrative produced by speaker 1 in several respects: Speaker 2 uses longer sentences than speaker 1 with more variation in their structure, speaker 2 provides more information than speaker 1 and explicitly mentions causal relations between events (as, e.g., marked by the causal connective *because*), and speaker 2 makes different referential choices than speaker 1 and uses fewer pronouns and more full noun phrases.

One reason for the observed differences between the narratives of the two speakers is that speaker 1 is a 6-year-old child, while speaker 2 is a 27-year-old adult. As an adult can be expected to have more linguistic experience than a child speaker and also is expected to possess more cognitive resources, this may be the reason for the longer and more elaborate sentences and the more explicit references in the narrative produced by speaker 2. However, suggesting a potential reason is not the same as providing an explanation. In particular, the observation that the two speakers differ in age does not tell us which aspects of children’s linguistic or cognitive abilities must develop further to result in more adult-like narrative productions.

In addition to variation among speakers, the two narratives also illustrate another type of variation, namely variation across tasks. When telling a story, speakers must introduce the characters in the story as referents in the linguistic discourse, maintain reference to these characters when talking about their actions, and occasionally shift the attention of the listener from one character to the other. These actions can be considered as separate tasks carried out by the same speaker. Crucially, these tasks are subject to different constraints. To introduce the two characters in the story, both speakers use a full noun phrase. On the other hand, to continue to refer to the two characters, the adult speaker uses pronouns as well as full noun phrases, whereas the child speaker only uses pronouns. As the referents of these pronouns may not always be uniquely identifiable for a listener, these pronouns may underspecify the intended meaning. So the adult speaker and the child speaker make similar referential choices on the task of introducing referents, but make different referential choices on the task of maintaining and shifting reference. This illustrates that there is an intricate interaction between a speaker’s linguistic and cognitive abilities and the properties of the task.

To investigate the interaction between linguistic constraints, cognitive constraints and task effects on referential choice, it is useful to combine psycholinguistic experimentation with computational modeling. Using computational modeling can help in teasing apart the different factors involved in referential choice and shed more light on the way they interact. This may contribute to our understanding of why speakers overspecify and underspecify their referring expressions and why different speakers do so to different degrees. Also, computational modeling may reveal how the speaker’s referential choices affect the listener. The paper will focus on the type of referring expression and *how* speakers refer (e.g., with a full noun phrase or a pronoun) and how listeners interpret such referring expressions. Although the speaker’s choice of *what* to refer to (e.g., whether to express a causal relation between two events or not, or whether to refer to the pirate or the knight) also is an important aspect of referential choice, this is beyond the scope of this paper.

## Computational Modeling in ACT-R

Computational modeling of language often has the practical goal of developing computational algorithms that can be used in natural language applications. Virtually all natural language generation systems contain a module for generating referring expressions (Mellish et al., 2006). Although the aim of these systems is to be practically useful, computational models for the generation of referring expressions are usually not evaluated in terms of their usefulness, but instead in terms of their human-likeness (see Krahmer and van Deemter, 2012, for a comprehensive survey). In particular, these computational models aim to mimic human performance such as reflected in the group results of behavioral studies or in the patterns found in a corpus of written texts. For example, Kibrik and colleagues (Kibrik et al., 2013) developed a computational model that, using classical machine learning algorithms, determines referential choice in discourse on the basis of multiple factors related to properties of the referent and the discourse context. As these factors and their weights are extracted from a corpus of newspaper articles, they pertain to general patterns of referential choice generated by multiple writers on multiple occasions, as opposed to the specific referential choices made by individual writers in particular situations. However, if speakers and writers show variation in their referential choices, the specific patterns produced by individual speakers or writers may differ from the general patterns observed at the group level. Furthermore, computational models of this type are usually evaluated on the basis of their similarity with human oﬄine referential choice only, rather than on the basis of human online referential processing as well. Also, they are generally concerned with either language production or language comprehension, but not both.

In addition to its use in natural language applications, computational modeling of language can also be useful for the development of psycholinguistic theories. Regarding reference, the aim of such computational models is to mimic human oﬄine as well as online referential processes. Computational modeling of language for psycholinguistic research makes it possible to assess the completeness of a theoretical account, forces the modeler to be precise, allows for the systematic manipulation of factors, and makes possible the generation of novel predictions. In this paper, we discuss a series of computational models of reference production and comprehension that have been implemented in the cognitive architecture ACT-R (Adaptive Control of Thought-Rational; Anderson et al., 2004; Anderson, 2007). Computational modeling in ACT-R has the additional advantage that ACT-R not only is a computational modeling environment, but also is a theory of human cognition in which detailed assumptions about cognitive processes have been implemented that are based on a range of data from psychological and neurocognitive experiments. As a consequence, ACT-R’s modeling environment constrains the computational models in such a way that the models are cognitively plausible and are consistent with what is currently known about human cognition.

ACT-R is a hybrid architecture that combines symbolic and subsymbolic structures and processes. Whereas the chunks of factual information and the if-then production rules of ACT-R are symbolic in nature, certain processes of ACT-R are subsymbolic. When more than one production rule can be applied, there will be competition among these rules. The production rule with the highest expected utility will be executed. This is a subsymbolic process that is computed on the basis of mathematical equations weighing the costs of executing the production rule against its benefits. Another process that is dependent on properties at the subsymbolic level is the retrieval of chunks from declarative memory. Whether and how fast a chunk is retrieved depends on its activation value, which is a function of its frequency, its recency of use, and its connections to other chunks in memory.

A fundamental property of ACT-R is the assumption that each operation of the model takes time to perform. Every retrieval of a fact from declarative memory and every execution of a production rule takes a certain amount of time. Hence, performance of the model is limited by the time available for the cognitive process. However, the total execution time of the cognitive process is not simply the sum of the durations of all constituting operations. This is because the different modules of ACT-R can operate in parallel, although each module by itself can only perform a single operation at a time. Thus, the duration of a cognitive process critically depends both on the timing of the serial processes within a module and on how the different modules interact. Furthermore, there is some random variation in the model, as the utilities associated with production rules and the activation values of chunks are noisy. Therefore, to provide specific time estimations for a cognitive process, simulations should be run with the computational model (Anderson et al., 2004).

An ACT-R model obtains higher processing efficiency and performs faster by means of the ACT-R learning mechanism of production compilation (Taatgen and Anderson, 2002). In production compilation, two existing production rules are integrated into one new production rule. Because fewer production rules are needed with this new single production rule than with the old two production rules, the result is faster and more automatic processing. Production compilation occurs when two existing production rules are repeatedly executed in sequence. Ultimately, as a result of production compilation, carrying out a cognitive task may not require retrieval of individual chunks from memory or execution of multiple production rules anymore, but may be done by a single general production rule.

The predictions of an ACT-R model can be tested by comparing the results of computational simulations of the ACT-R model on a specific cognitive task with the results of human participants carrying out the same task. The output of a simulation in ACT-R consists of quantitative measures of performance on the task and estimates of the time it takes to perform the task. Each simulation of the model simulates the performance of an individual participant on a task. By offering different amounts of training, the model can also simulate the performance of individual children of different ages (van Rij et al., 2010). Due to the random variation present in the model, performance of the model differs slightly during each run. Thus, ACT-R models are non-deterministic. By running an ACT-R model several times on the experimental items of a linguistic task, a dataset is obtained that can be compared to – and analyzed in the same way as – the dataset obtained from a group of human participants on the same task.

Because of the cognitive constraints placed on computational models in ACT-R, ACT-R can shed more light on the cognitive processes involved in language and communication (cf. Taatgen and Anderson, 2002; Budiu and Anderson, 2004; Lewis and Vasishth, 2005; Reitter et al., 2011; Guhe, 2012). In particular, ACT-R’s assumptions regarding the duration of cognitive operations allow us to make precise predictions about the time course of language processing. Furthermore, ACT-R makes it possible to integrate linguistic analyses of referential choice in the model, implement the opposite processes of language production and language comprehension in one and the same model, and describe the development and processing of perspective taking in language without additional assumptions. Cognitive modeling in ACT-R may therefore reveal the mechanisms underlying the observed variation in speakers’ and listeners’ referential choices.

## Cognitive Modeling of Reference Production and Comprehension

In a series of studies (Hendriks et al., 2007; van Rij et al., 2010, 2013; van Rij, 2012; Vogelzang et al., 2015), computational models have been implemented within the cognitive architecture ACT-R to simulate the production and comprehension of referring expressions by adults and children. These computational simulations focused on the type of referring expression (definite noun phrase, overt pronoun or null pronoun) in production and on the identification of the referent in comprehension. The outcomes of these simulations were compared to existing data and further simulations were run to generate new predictions. The details of the various ACT-R models of reference are presented below. As these models are based on the same principles, we will refer to them as the ACT-R model of reference, only mentioning differences between the models when relevant. Following the presentation of the ACT-R model of reference, it is discussed how the model explains individual variation in reference production and comprehension and how the model generates novel predictions that can be tested empirically.

**FIGURE 2:**
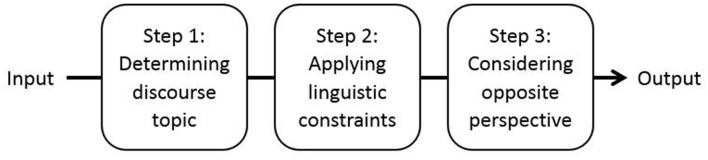
**Performance of the ACT-R model of reference.** Performance of the model proceeds in three steps. In production, the input is a meaning and the output is the optimal form for expressing this meaning. In comprehension, the input is a form and the output is the optimal meaning assigned to this form.

Performance of the ACT-R model of reference proceeds in three steps (see **Figure 2**): (1) determining the topic of the linguistic discourse on the basis of general memory principles, (2) applying the linguistic constraints that underlie the choice and interpretation of referring expressions, and (3) considering the opposite perspective in communication, which provides internal feedback to the model on the correctness of the referential choice.

These three steps are discussed in more detail below. Particular emphasis is placed on the cognitive principles and mechanisms that are implemented in the model and that may be relevant for reference production and comprehension.

### Step 1: Determining the Current Discourse Topic

The first step of the ACT-R model of reference (see, e.g., van Rij et al., 2013) consists of determining the current discourse topic. Using the general memory principles of ACT-R, the model incrementally builds a (simplified) representation of the linguistic discourse during online processing. Each discourse referent that is encountered is represented as a chunk in declarative memory that has a certain amount of activation. Within ACT-R, the activation of a chunk depends on its frequency of use and the recency of the last retrieval of the chunk. The more frequently the chunk is used, or the more recent its last retrieval, the higher its activation. The activation of a chunk decays with time, but is increased when the chunk is retrieved again. The discourse referent with the highest level of activation in declarative memory is taken to be the current discourse topic. This allows the model to use gradient information about the activation of referents for making discrete decisions about the linguistic effects of discourse topicality in the next step of the model.

In addition to this mechanism of *base-level activation*, ACT-R also has a mechanism of *spreading activation*, that can temporarily increase the activation of a chunk. Spreading activation reflects the usefulness of a chunk in a particular context: chunks that are currently being processed spread activation to connected chunks in declarative memory. In van Rij et al.’s (2013) model, the subject of the previous sentence is temporarily stored as goal-relevant information and therefore spreads activation to connected chunks. This reflects the observation that the subject of the previous sentence is likely to be the current discourse topic (e.g., Grosz et al., 1995). Because the referent that was mentioned as the subject of the previous sentence becomes more activated in comparison to other referents due to spreading activation, the model will more often select this referent as the discourse topic.

Building a representation of the discourse requires access to memory resources, which can be different for different individuals. ACT-R does not have a separate working memory (WM) component. However, one of the ways to model WM effects in ACT-R is through individual differences in spreading activation (van Rij et al., 2013). The amount of spreading activation determines the ability to maintain goal-relevant information, and differences in the total amount of spreading activation account for individual differences in WM capacity (Daily et al., 2001). Hence, the effects of WM capacity on discourse processing can be modeled as resulting from differences in the ability to maintain goal-relevant information pertaining to the subject of the previous sentence (van Rij et al., 2013). In the ACT-R model of reference, a high WM capacity gives rise to a large amount of spreading activation of the chunk representing the subject of the previous sentence. This results in this previous subject being a determining factor in the selection of the discourse topic. In contrast, a low WM capacity only gives rise to a small amount of activation, resulting in no effect at all of the subject of the previous sentence on the selection of the discourse topic. In the latter case, frequency and recency will be the main determinants of the discourse topic.

The mechanism of base-level activation in combination with spreading activation implements the effects of the preceding linguistic discourse on the prominence, or accessibility, of discourse referents. Referents are more accessible if they are more frequently referred to, more recently referred to, or mentioned as the subject of the preceding sentence (cf. Givón, 1983; Ariel, 1988, 1990; Grosz et al., 1995; Arnold, 2010). Furthermore, influences of WM capacity on the selection of the discourse topic are predicted.

### Step 2: Applying Linguistic Constraints on Referential Choice

The second step of the ACT-R model of reference consists of the application of linguistic constraints that restrict the choice and interpretation of referring expressions. These linguistic constraints and the way they interact are taken from Optimality Theory (Prince and Smolensky, 2004) and from theoretical analyses of referential choice in this linguistic framework. Constraints in Optimality Theory differ from rules in rule-based linguistic frameworks in that these constraints are formulated as general as possible and hence can be in conflict. Crucially, the constraints differ in strength and are violable. If two constraints are in conflict and cannot be satisfied both, the stronger constraint is satisfied at the cost of violating the weaker constraint. A second difference between linguistic constraints and linguistic rules is that, whereas linguistic rules are input-oriented, linguistic constraints are output-oriented. Rules apply if the input conditions are met. Constraints, on the other hand, apply if the output has particular features. For example, a constraint prohibiting the use of pronouns will apply if a potential output contains a pronoun. This property of constraints allows Optimality Theory to explain mismatches – that is, asymmetries – between production and comprehension in child language (Smolensky, 1996; Hendriks, 2014), as is explained below.

To produce or interpret a referring expression, the ACT-R model evaluates potential outputs for a particular input. In production, the input meaning is given and potential forms for expressing this meaning compete. On the basis of the constraints of the grammar, the optimal form for expressing the input meaning is selected from a set of competing forms. The optimal form is the form that satisfies the constraints of the grammar best. Whereas in production the input consists of a meaning and the output is the optimal form for this meaning, in comprehension the input consists of the form to be interpreted and the output is the optimal meaning for this form. Determining the optimal meaning in comprehension is subject to the same hierarchy of constraints as in production. Thus, production and comprehension are guided by the same grammar and only differ in the direction of optimization (from input meaning to optimal form versus from input form to optimal meaning).

**FIGURE 3:**
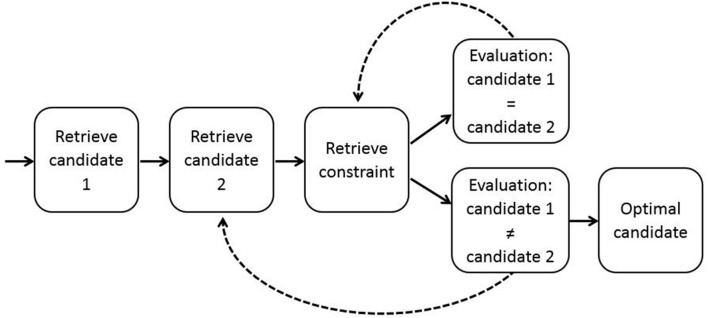
**Selection of the optimal candidate in the ACT-R model of reference.** This process of optimization occurs in Steps 2 and 3. After retrieval of two candidates and a constraint, the two candidates are evaluated on the basis of the constraint. This procedure is applied iteratively, retrieving new candidates and new constraints until the optimal candidate is found or time is up. Adapted from Hendriks et al. (2007).

In the ACT-R model, candidate forms, candidate meanings and linguistic constraints are implemented as chunks in declarative memory (see Misker and Anderson, 2003, for an alternative approach to combining ACT-R with Optimality Theory). Rather than determining the optimal candidate by simultaneously comparing all candidate outputs with respect to the complete hierarchy of constraints, as is assumed in theoretical work in Optimality Theory (Prince and Smolensky, 2004), the ACT-R model compares only two candidates at a time, starting with the candidates with the highest activation. Each of these two candidates is evaluated on the basis of only one constraint at a time, starting with the strongest constraint. If one of the two candidates satisfies this constraint and the other does not, this other candidate is discarded and a new candidate is retrieved from memory. If the two candidates both violate or satisfy the constraint, a next constraint is retrieved. The two candidates are then evaluated on the basis of this next constraint. By iteratively applying this procedure (see **Figure 3**), given sufficient time all candidates can be evaluated with respect to all constraints. The optimization procedure terminates if an optimal candidate has been found or if time is up. In the latter case, one of the two candidates under consideration is selected at random.

Two linguistic constraints that have been argued to be relevant for the production and comprehension of referring expressions in discourse (e.g., Hendriks et al., 2008) are Referential Economy (referentially less informative forms such as pronouns are preferred to referentially more informative forms such as full noun phrases) and ProTop (pronouns refer to the discourse topic). The former constraint is theoretically modeled in Optimality Theory as a family of constraints of differing strengths prohibiting referring expressions. As the constraint prohibiting full noun phrases is stronger than the constraint prohibiting pronouns, it is better to use a pronoun than to use a full noun phrase.

On the basis of these two constraints, an overall preference is predicted for producing pronouns, even for referents that are not highly prominent in the discourse. Furthermore, it is predicted that all pronouns are interpreted as referring to the discourse topic, that is, the most prominent referent in the discourse. This asymmetric pattern in the production and comprehension of anaphoric pronouns is consistent with the literature on children’s use and interpretation of pronouns in discourse (e.g., Karmiloff-Smith, 1985; Song and Fisher, 2005). For example, Karmiloff-Smith (1985) notes that, in narrative production, 4-year-olds produce strings of pronouns that at times refer to the main character of the story and at other times to the subsidiary character, thus making reference ambiguous for the listener. On the other hand, 3-year-olds’ comprehension of pronouns already depends in an adult-like way on the prominence of the referents in the linguistic discourse (Song and Fisher, 2005). So, in child language correct production of anaphoric pronouns seems to lag behind their correct comprehension. This particular asymmetry between production and comprehension is predicted by the two constraints mentioned above.

In contrast to children, adults do not show an overall preference for producing pronouns, regardless of the discourse context. Instead, their referential choices in production match their referential choices in comprehension. This is the motivation for the third step of the model, which further restricts adults’ production of anaphoric pronouns by means of perspective taking.

For the production and comprehension of pronouns in syntactic binding environments, such as *her* in the sentence “*Goldilocks washed her*,” the additional syntactic constraint Principle A is relevant. This constraint requires a reflexive to be bound within its clause (cf. Chomsky, 1981). That is, it requires *herself* in the sentence “*Goldilocks washed herself*” to be coreferential with the local subject *Goldilocks*. As Principle A is stronger than the constraint from the constraint hierarchy Referential Economy that prefers reflexives to pronouns, the two constraints together predict that local binding is expressed by reflexives and that reflexives are interpreted as being locally bound. Furthermore, these constraints predict that unbound referents are expressed by pronouns, as Principle A does not allow reflexives to appear unbound. This pattern is indeed observed in English-speaking children and adults. However, English-speaking children differ from adults in their interpretation of pronouns in syntactic binding environments. For children, such pronouns are ambiguous and can receive a bound as well as an unbound interpretation. That is, they take *her* in “*Goldilocks washed her*” to be able to refer to Goldilocks too. This asymmetric pattern is again predicted by the constraints and is generally known as the Delay of Principle B Effect, referring to the delayed development of object pronouns compared to reflexives in languages such as English and Dutch (Chien and Wexler, 1990; van Rij et al., 2010). In contrast to the asymmetry with anaphoric pronouns discussed above, in case of object pronouns in syntactic binding environments correct comprehension surprisingly lags behind correct production (De Villiers et al., 2006; Spenader et al., 2009). Because the interpretation of object pronouns is not restricted by syntactic constraints, object pronouns are allowed to be coreferential with the local subject. However, for adults this is not true. Again, the third step of the model is needed to further restrict adults’ interpretation of pronouns.

The second step of the ACT-R model of reference crucially relies on linguistic knowledge. This implies that cross-linguistic differences in referential choice must receive their explanation in this part of the model. For example, the fact that in languages such as English sentences must always have a subject, whereas in languages such as Italian pronominal subjects can be dropped, can be explained by a different ranking of the same two constraints (Grimshaw and Samek-Lodovici, 1998). The availability of the additional possibility for expressing the subject in Italian as a null pronoun not only influences the distribution of overt pronouns, but may also influence the way these overt pronouns are interpreted (Vogelzang et al., 2015). As the three steps of the model are closely connected, these cross-linguistic differences in the constraint hierarchy and the inventory of linguistic forms are expected to also affect the other steps of the model.

### Step 3: Considering the Opposite Conversational Perspective

The third step of the ACT-R model of reference is the consideration of the opposite perspective in communication. After an initial choice has been made by the model in Step 2, the opposite communicative perspective is taken to verify whether this initial choice is also optimal from the opposite perspective. In production, the model first takes the perspective of the speaker to select a referring expression, and next takes the opposite perspective of a listener to check whether this referring expression is understandable for a hypothetical listener in the (speaker’s representation of the) current linguistic discourse. Likewise, in interpretation, the model first takes the perspective of the listener to select a referent for the referring expression that is encountered, and next takes the opposite perspective of a speaker to check whether a hypothetical speaker would indeed have chosen this expression to refer to the selected referent in the (listener’s representation of the) current linguistic discourse.

**FIGURE 4:**
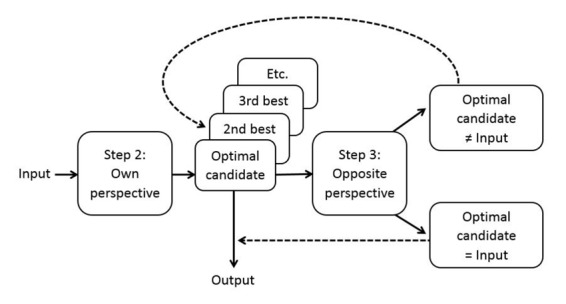
**Perspective taking in the ACT-R model of reference.** Perspective taking involves selection of the optimal candidate from the language user’s own perspective (Step 2), followed by selection of the optimal candidate from the opposite communicative perspective (Step 3). The output is the best candidate in Step 2 that produces the input again in Step 3.

This mechanism of perspective taking is a serial implementation of the algorithm of bidirectional optimization in Optimality Theory (Blutner, 2000). Bidirectional optimization considers all pairs of linguistic form and meaning simultaneously and identifies the optimal pairs. It has the effect that if a form or meaning already is part of an optimal form-meaning pair, its use is blocked for another form-meaning pair. In the ACT-R model, bidirectional optimization is implemented as a serial process of perspective taking, starting with optimization from the language user’s own perspective followed by optimization from the opposite perspective (Hendriks et al., 2007). This two-step process of perspective taking proceeds incrementally. That is, perspective taking is not postponed until the end of the sentence and also does not consider all pairs of form and meaning in one step (as in Blutner’s bidirectional optimization algorithm), but rather is applied online and only considers two possibilities at a time. The extra step of optimization from the opposite perspective proceeds in the same way as optimization from the own perspective (as shown in **Figure 3**), after which the output of the extra step of optimization is compared to the input of the initial step of optimization (see **Figure 4**). If the output of the extra step of optimization (Step 3) differs from the input of the initial step of optimization (Step 2), the initially selected form or meaning is discarded and the next best form or meaning is taken as the input to the extra step of optimization. This process is repeated iteratively until output and input match or until time is up.

The mechanism of perspective taking thus generates internal feedback for the model. This feedback takes the form of a match or mismatch between the form-meaning pair resulting from optimization from one’s own perspective and the form-meaning pair resulting from optimization from the opposite conversational perspective. A match results in an update of the parameters associated with the production rules that were used, increasing the chances that these production rules are used again next time. Mismatches have the effect that forms whose meaning is not recoverable for a listener and interpretations that are not expressed with the heard form by a speaker are blocked.

Obviously, the extra step of perspective taking takes additional time. Therefore, performing both steps (the step of the initial selection of the form or meaning and the additional step of perspective taking) during online production and comprehension requires sufficient processing speed (van Rij et al., 2010). Initially, the model is unable to complete both steps, because this takes too much time. As a consequence, the model is only able to complete the step of the initial selection of a form or meaning and does not take into account the opposite communicative perspective. In ACT-R, processes become more efficient with linguistic experience due to the ACT-R learning mechanism of production compilation (Taatgen and Anderson, 2002). By frequently performing the processes of reference production and comprehension, the relevant production rules are repeatedly carried out in sequence. Production compilation reduces the number of production rules required for processing and hence reduces the amount of time needed for processing. As the model thus gradually gains more processing speed, the model will become more likely to take into account the opposite perspective. Eventually, through the mechanism of production compilation the two-step process of perspective taking may turn into a one-step selection process. Thus, it is predicted that the ability to use perspective taking in real-time conversation is dependent on sufficient processing speed, which in turn is dependent on linguistic experience. Linguistic experience increases with age as well as with frequency of the referring expression: the older the child and the more frequent the referring expression in the language input to the child, the more experience the child can be expected to have with the referring expression.

As perspective taking requires an awareness that speakers may possess different knowledge and make different choices than listeners, the ability of perspective taking in language may be related to the development of a Theory of Mind (ToM). ToM refers to the cognitive capacity to attribute mental states, such as beliefs, desires and intentions, to oneself and others and to understand that the mental states of others may differ from one’s own mental states (Premack and Woodruff, 1978). First-order ToM, the capacity to understand what another person thinks, typically emerges in children around the age of 3 or 4 in explicit false-belief tasks (e.g., Wimmer and Perner, 1983). Second-order ToM, which builds on first-order ToM and is the capacity to understand what another person thinks about what yet another person thinks, emerges several years later, around the age of 6 (Perner and Wimmer, 1985). Because of its assumed relation to ToM development, it is conceivable that perspective taking in language only fully develops after age 3 or 4.

**FIGURE 5:**
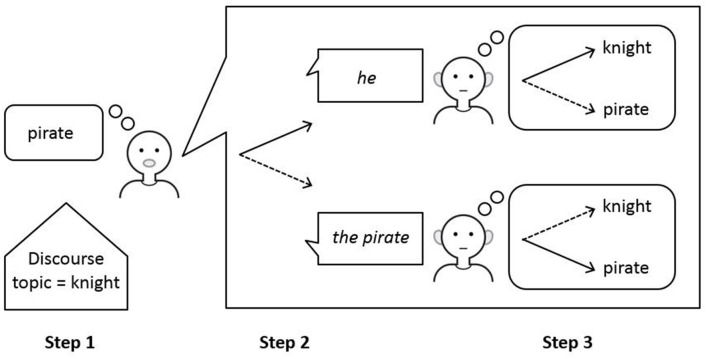
**Production of referring expressions in the ACT-R mode of reference.** A speaker wishing to refer to a referent that in Step 1 was found not to be the current discourse topic (e.g., the pirate in the final picture of the story in **Figure 1**) preferably uses the pronoun *he* to refer to this referent (indicated by the solid arrow in Step 2). Taking into account the perspective of the listener in Step 3 will reveal that *he* is best interpreted as the current discourse topic (indicated by the solid arrow in the top picture in Step 3). As this referent (knight) is different from the intended referent (pirate), the pronoun is blocked as a potential form and a full noun phrase must be selected. Adapted from Hendriks (2014).

The final step of perspective taking is crucial for the mature choice between a pronoun and a full noun phrase (see **Figure 5**). If the model is only able to complete the process of initial selection of a form or meaning and is unable to complete the next process of perspective taking, the model will produce pronouns all the time for expressing anaphoric reference. It will do so even for referents that are not the discourse topic. On the other hand, if the model is able to take into account the opposite perspective of the listener, pronouns are blocked for referents that are not the discourse topic. As a result, the model will restrict its use of pronouns to referents that are the discourse topic. For other discourse referents, the model will select a full noun phrase.

**FIGURE 6:**
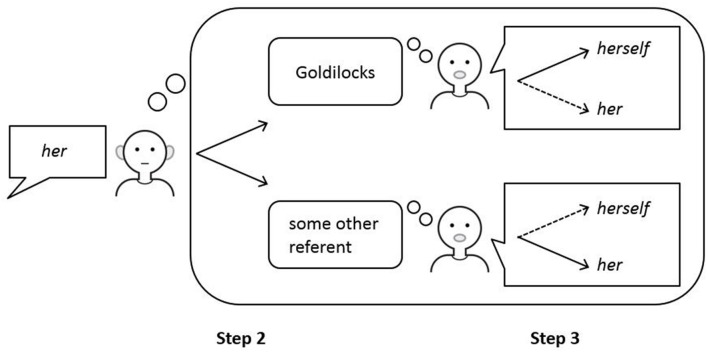
**Comprehension of referring expressions in the ACT-R model of reference.** A listener hearing the sentence “*Goldilocks washed her*” in a context that provides no referential bias (hence, Step 1 is omitted here) may select Goldilocks or some other referent as the antecedent of the pronoun *her* (indicated by the two solid arrows in Step 2) and will make a random choice. If Goldilocks is selected as the antecedent of *her*, taking into account the perspective of the speaker in Step 3 will reveal that reference to Goldilocks is best expressed by the reflexive *herself* (indicated by the solid arrow in the top picture in Step 3). As this form is different from the heard form *her*, the referent Goldilocks is blocked as a potential antecedent for the pronoun *her* and some other referent must be selected. Adapted from Hendriks (2014).

Perspective taking is also crucial for the mature comprehension of pronouns in syntactic binding environments (see **Figure 6**). If the model is unable to complete the process of perspective taking, pronouns in object position in languages such as English will remain ambiguous and can be interpreted as referring to the local subject. However, if the model is able to take into account the opposite perspective of the speaker, this bound interpretation will be blocked and pronouns in object position will be interpreted as being non-coreferential with the local subject.

Thus, perspective taking in production and comprehension has the effect of avoiding misunderstanding between speaker and listener. Note that avoiding misunderstanding crucially differs from avoiding ambiguity: producing referentially ambiguous expressions such as pronouns is permitted by the model, as long as the ambiguity does not result in misunderstanding between speaker and listener in the given discourse context. Our view of perspective taking as a crucial step in the production and comprehension of particular linguistic expressions that is nevertheless still difficult for children, may contribute to the current debate about the role of perspective taking in language. Various positions have been put forward in this debate: that language users are initially egocentric and only adjust their perspective when circumstances demand (e.g., Horton and Keysar, 1996; Keysar et al., 2003), that language users are initially egocentric because they are unable to fully discount their own perspective (Barr, 2008), and that perspective taking is one of many cues in language processing that is used early on (e.g., Brown-Schmidt et al., 2008). As pointed out by Brown-Schmidt (2009), empirical findings have been equivocal about the online use of perspective taking in language processing, so one of the main challenges for models of perspective taking in language is to account for why perspective taking sometimes constrains online processing and sometimes does not.

Because of its direct appeal to cognitive capacities such as WM, processing speed and ToM, which can vary among individual speakers and listeners, the ACT-R model of reference seems particularly suited to explain and predict different patterns of variation in reference production and reference comprehension. In the next section, the model’s predictions are discussed for speakers’ underspecification and overspecification of referring expressions and for listeners’ incorrect or overly liberal interpretation of referring expressions.

## Explaining and Predicting Individual Variation

### Underspecification of Referring Expressions

As mentioned above, children prefer to use pronouns over full noun phrases, even when referring to referents that are not the discourse topic (e.g., Karmiloff-Smith, 1985). As these pronouns underspecify their referent, they may cause misunderstanding for a listener. For this reason, adult speakers generally use full noun phrases when referring to a referent that is not the discourse topic.

Performing simulations with the ACT-R model of reference, we can investigate the effects of cognitive factors on reference production and comprehension by manipulating features of the model. van Rij (2012, Chap. 3) modeled the production of referring expressions in a linguistic discourse and investigated the effects of WM on the performance of the model. The performance of two variants of this model was compared: a model with a low WM capacity and a model with a high WM capacity (implemented by spreading activation). In the simulations run with the models, the models were presented with stories of five sentences each about two referents of the same gender, similar to the narratives produced by children and adults for the picture story in **Figure 1**. Each story started with the first referent being the subject of the sentence and hence the topic of the discourse. Halfway through the story, the topic shifted from the first to the second referent by making this second referent the subject of that sentence and the next one. After having presented the model with this linguistic discourse, the task of the models was to produce a referring expression to refer back to the first referent, which at that point in the discourse was not the topic anymore.

The low WM capacity model produced underspecified pronouns to refer back to the first referent in 86% of the cases, and produced explicit full noun phrases in the remaining 14% of the cases. In contrast, the high WM capacity model produced pronouns in only 11% of the cases and full noun phrases in the large majority of cases, namely 88%. The performance of the low WM capacity model reflects the performance of the children in the study by Hendriks et al. (2014). They tested 4- to-7-year-old Dutch-speaking children on a narrative elicitation task based on picture stories consisting of six pictures each, such as in **Figure 1**. The stories elicited a topic shift from the first referent (the pirate) to the second referent (the knight) halfway through the story. To re-introduce the first referent in the final picture of the story, which is not the discourse topic anymore at the moment of re-introduction, the children in the study produced pronouns in 62% of the cases and full noun phrases in 38% of the cases. This preference for pronouns was in accordance with the performance of the low WM capacity model. In contrast, the performance of the high WM capacity model reflects the performance of the young Dutch adults in the same study. These adults produced pronouns in only 9% of the cases and produced full noun phrases in 91% of the cases.

The model’s prediction that WM is a crucial factor in the choice between a pronoun and a full noun phrase is supported by experimental evidence from narrative elicitation studies with various populations. Hendriks et al. (2014) found a positive correlation between the use of full noun phrases by the children and their scores on an auditory memory task (word repetition): the higher the children’s memory scores, the more often they used a full noun phrase to re-introduce the first referent. A similar effect of WM (this time measured by an n-back task) was found in a study with children with autism, children with ADHD and typically developing children in the age range between 6 and 12 years old who were tested on the same narrative elicitation task (Kuijper et al., 2015). In addition to the effect of WM, Kuijper et al. also found an effect of second-order ToM: the higher the children’s scores on second-order ToM (as measured by a false-belief task), the more full noun phrases they used for referring back to the first referent.

Inspecting the ACT-R model’s performance allows us to more closely examine the reasons for selecting an underspecified pronoun, in particular for the way WM and processing speed influence this choice. Of the 86% of cases in which the low WM capacity model produced a pronoun, in two third of these cases (57%) this is caused by a low amount of spreading activation (van Rij, 2012, p. 64). Due to its low amount of spreading activation, the low WM capacity model does not take into account the grammatical roles of referents in the local discourse. It only relies on frequency and recency of mentioning. Hence, it shows a strongly reduced preference for selecting the subject of the previous sentence (the second referent) as the discourse topic. As a result, the low WM capacity model is not very accurate in determining the discourse topic and will often select the first referent. If the model incorrectly selects the first referent as the discourse topic, a pronoun is the optimal form for re-introducing this first referent, both according to the linguistic constraints and after considering the opposite perspective. However, for a listener who has access to the preceding linguistic discourse and correctly selects the second referent as the discourse topic, this pronoun will be interpreted as referring to the second referent. Thus, the use of a pronoun after a topic shift will result in misunderstanding.

In addition to low WM capacity, the ACT-R model reveals a second reason for using an underspecified pronoun after a topic shift, namely insufficient speed of sentence processing. Of the 86% of cases in which the low WM capacity model produced a pronoun, in one third of these cases (29%) this is caused by insufficient processing speed (van Rij, 2012, p. 64). The linguistic constraints lead the model to have a general preference for using a pronoun. Only if the model succeeds in taking into account the listener’s perspective to check the recoverability of the initially selected form will the model block the use of a pronoun and select a more explicit full noun phrase instead (see **Figure 5**). As the completion of the process of perspective taking is dependent on sufficient processing speed (van Rij et al., 2010), insufficient processing speed results in the use of underspecified pronouns. As we saw above, also less advanced ToM abilities are related to the use of underspecified pronouns (Kuijper et al., 2015). It is conceivable that children with less advanced ToM abilities are slower in perspective taking, thus having insufficient time to complete the process of perspective taking during online sentence processing.

So, based on simulations of the ACT-R model, it can be argued that the mature use of pronouns requires sufficient WM capacity (which increases through maturation) and sufficient processing speed (which increases through linguistic experience). The observed differences between children and adults in their production of referring expressions can thus be explained by individual differences in their WM capacity and processing speed.

In addition to explaining existing data regarding reference production, the ACT-R model also generates novel predictions, that can be tested in subsequent experiments. For example, adults with a low WM capacity but sufficient processing speed are predicted to frequently use a pronoun to refer to a referent which they incorrectly take to be the discourse topic, just like children. This follows from their expected failure to use grammatical role information from the previous sentence in determining the discourse topic. Indeed, the elderly adults with a mean age of almost 80 in the study of Hendriks et al. (2014), whose average score on the memory task was significantly lower than that of the young adults, but who can be expected to still have sufficient processing speed, produced pronouns to re-introduce the first referent in almost half of the cases (47%). An indication that the elderly adults’ production of underspecified forms is due to their low WM capacity, and is not caused by their failure in perspective taking, is the observation that they produced significantly more full noun phrases when re-introducing the first referent in the sixth picture than when referring to the second referent in the fifth picture. This suggests that elderly adults do take the listener into account when re-introducing the first referent (Hendriks et al., 2014), and thus possess sufficient processing speed.

### Overspecification of Referring Expressions

Another novel prediction of the ACT-R model of reference, one that has not been tested yet, is that particular linguistic discourse contexts lead speakers to produce overly specific referring expressions because of insufficient WM capacity. That is, in these discourse contexts speakers with insufficient WM capacity but sufficient processing speed are expected to use a full noun phrase to refer to the discourse topic, although a pronoun would have sufficed.

Insufficient WM capacity can occur for several reasons. Speakers may have insufficient WM capacity due to age or cognitive deficits. Alternatively, they may have insufficient WM capacity available because their WM is overloaded by other cognitive processes. This can happen when a speaker has to carry out two or more tasks simultaneously. In ACT-R, the effect of high cognitive load is similar to the effect of low WM capacity (van Rij et al., 2013): due to the mechanism of spreading activation, goal-relevant information spreads activation to other chunks in declarative memory. If the number of sources from which activation is spread increases, the amount of spreading activation received by individual chunks decreases, because the total amount of spreading activation is fixed. In a situation of high cognitive load, more information needs to be maintained in an activated state. Thus, more sources spread the fixed amount of spreading activation. As a result, the subject of the previous sentence spreads less activation to the discourse referent associated with the subject and hence this referent is less likely to be selected as the discourse topic.

The particular linguistic discourse contexts that are predicted to lead speakers to overspecify their referring expressions are contexts in which the discourse topic shifts from a more frequently or more recently mentioned referent to a less frequently or less recently mentioned referent. In such discourse contexts, a speaker with sufficient WM capacity will signal the topic shift (e.g., by expressing the new topic as a full noun phrase in subject position) and then continue to refer to this new topic using a pronoun. A speaker with insufficient WM capacity, on the other hand, may continue to refer to this new topic by using a full noun phrase. This is because the lack of sufficient WM capacity causes the speaker to rely less on grammatical role information from the previous sentence and more on frequency and recency of mentioning when determining the discourse topic. If the new topic is a less frequently or less recently mentioned referent, the speaker may incorrectly assume that this referent has not been established as the discourse topic yet and use a full noun phrase to refer to this referent.

Thus, it is predicted that young adult speakers carrying out an additional task that taxes their WM will produce more overspecified referring expressions for reference to the new topic immediately after a topic shift. The speaker will continue to use such overspecified referring expressions until the accessibility of the new discourse topic has increased by frequent mentioning or recency. Some suggestive evidence in favor of this prediction comes from the use of referring expressions by adult learners of a second language. Speaking a foreign language generally requires more cognitive resources, including WM, than speaking the native language (e.g., Linck et al., 2013). In a study investigating narratives produced by adult intermediate and advanced learners of French and English when retelling a silent cartoon movie, the adult second-language learners were found to overspecify referring expressions compared to native speakers of the two languages and to use definite noun phrases where pronouns could be used (Leclercq and Lenart, 2013). These overspecifications particularly occurred when the speakers had to re-introduce the second character. As the two characters appeared to be of a different gender to many of the participants, a pronoun would have sufficed. Although Leclercq and Lenart explain the overspecification of referring expressions in second-language learners as a conscious risk-avoiding strategy, these overspecifications may very well be the unconscious effects of insufficient WM capacity.

In addition to variation in production, in particular with respect to overspecification and underspecification, the ACT-R model also explains variation in comprehension. Below, we discuss some of the variation observed in adults’ and children’s interpretations of pronouns.

### Incorrect Interpretation of Referring Expressions

Adults who have less WM capacity available because their WM is taxed by an additional task are also more likely to select an incorrect referent for a pronoun. In particular, they are predicted to show difficulty comprehending a topic shift. As they are less likely to use grammatical role information from the preceding sentence due to the low amount of spreading activation, they will solely rely on frequency and recency of mentioning of the referents. In case of a topic shift, this will often result in selection of the incorrect referent as the discourse topic and hence as the antecedent of the pronoun.

In a dual-task experiment, van Rij et al. (2013) tested this prediction of the ACT-R model. Adult participants had to perform two tasks at the same time. The linguistic task was a pronoun comprehension task. The additional task was the memorization of a series of digits. While memorizing either three digits (low cognitive load condition) or six digits (high cognitive load condition), participants read short stories consisting of four sentences. The stories featured two referents of the same gender, which were only referred to with proper names. The final sentence started with a potentially ambiguous subject pronoun that could in principle refer to both referents (e.g., “*He has played soccer for twenty years*”). Following the story, participants received a comprehension question, which asked for the referent of the pronoun. They received two types of stories: stories with and stories without a topic shift. These two types of stories only differed in the grammatical roles of the referents in the second and third sentence (subject or non-subject). In the topic-shift stories, the topic was shifted from the most frequently mentioned referent to the other referent by making this other referent the subject of the second and third sentence. In the non-topic-shift stories, the most frequently mentioned referent remained the subject of the sentence throughout the story. After answering the comprehension question, participants had to type in the digits that were presented to them before the start of the story. Each participant was tested in both cognitive load conditions.

As predicted, adults less often selected the subject of the previous sentence as the referent of the pronoun in the high cognitive load condition than in the low cognitive load condition. Instead, they selected the most frequently mentioned other referent. This effect of cognitive load was limited to stories with a topic shift and did not affect stories without a topic shift, which was in line with the predictions of the model. Thus, adult listeners more often assign an incorrect interpretation to an anaphoric pronoun under high cognitive load and select the most frequent referent in the discourse, instead of the linguistically most prominent referent.

### Overly Liberal Interpretation of Referring Expressions

The predictions discussed above mainly concerned WM capacity. An exception was the prediction that, in addition to low WM capacity, also insufficient speed of sentence processing results in the production of underspecified pronouns after a topic shift. Here, we discuss another prediction of the ACT-R model concerning processing speed, namely the prediction that insufficient processing speed results in an overly liberal interpretation of object pronouns in syntactic binding environments. Note that in the case of object pronouns, low WM capacity is predicted not to have an effect, as the correct interpretation of object pronouns is independent of the linguistic discourse.

As mentioned above, to interpret the object pronoun *her* in the sentence “*Goldilocks washed her*” and to restrict its interpretation to a referent that is not the local subject, a listener must take into account the perspective of the speaker. Taking into account the opposite perspective in addition to one’s own perspective is expected to take more time than only considering one’s own perspective, as the process of perspective taking is modeled in the ACT-R model of reference as two consecutive processes of optimization (Hendriks et al., 2007). Therefore, sufficient processing speed is needed to complete the process of perspective taking. Processing speed is increased by the ACT-R learning mechanism of production compilation, which depends on linguistic experience. Children may not have sufficient processing speed yet to be able to complete the process of perspective taking within the alotted time. However, they may be able to take into account the opposite perspective when given more time for interpretation.

In the ACT-R model, new words arrive at a fixed rate. This rate cannot be influenced by the listener. Therefore, time for interpretation of a word is limited to the time until the arrival of the next word. If the time until the next word is increased, children have more time for the interpretation of a sentence-internal pronoun and may be able to complete the process of perspective taking more often. van Rij et al. (2010) carried out a picture verification task with 4- to 7-year-old Dutch children to test this prediction. The children received sentences such as “*The bear is tickling him with a feather*” and had to say whether the sentence matched an accompanying picture or not. As the pronoun occurs mid-sentence, time for interpretation is limited to the arrival of the next word. Half of the sentences were presented to the child at a normal speech rate and the other half at a slower speech rate of 2/3 of normal speech rate. In the slower speech rate condition, the children had more time for the interpretation of the pronoun due to the extra time between words.

van Rij et al. (2010) found that, if children displayed the Delay of Principle B Effect, slowing down the speech rate improved their comprehension of pronouns. In contrast, slow speech rate had a negative effect on their (already adult-like) comprehension of reflexives. These selective beneficial effects of slowed-down speech support the assumption implemented in the ACT-R model that the mature interpretation of object pronouns requires perspective taking. It is also consistent with the view of perspective taking as an online and local process, rather than a pragmatic and end-of-sentence process, as it is dependent on sufficient processing speed during online sentence processing.

Based on these outcomes, it is further predicted that the mature comprehension of object pronouns is related to advanced ToM abilities, in the same way that avoiding to produce underspecified pronouns after a topic shift is related to advanced ToM abilities, as both processes are hypothesized to require perspective taking (see **Figures 5** and **6**). This contrasts with the mature comprehension of reflexives and the mature production of pronouns in topic continuation situations, which are expected not to be dependent on the additional step of perspective taking, as the linguistic constraints already lead to the correct output in these cases (Hendriks, 2014).

Another prediction that can be experimentally tested is that features of the linguistic discourse influence the oﬄine interpretation of object pronouns of listeners with low processing speed, but not of listeners with high processing speed. Perspective taking generates internal feedback, which restricts the interpretation of object pronouns and makes this process less dependent on the discourse (see **Figure 6**). As perspective taking depends on processing speed, reduction of the influence of the discourse also depends on processing speed. Thus, it is expected that the linguistic discourse influences children’s oﬄine interpretation of object pronouns, but not adults’ (although it may influence adults’ online processing). In particular, if the *correct* antecedent of the pronoun is the most frequently and most recently mentioned referent, children will be biased toward the correct antecedent in Step 2, whereas if an *incorrect* antecedent is the most frequently and most recently mentioned referent, children will be biased toward the incorrect antecedent in Step 2. Without the internal feedback provided by perspective taking in Step 3, children will stick to their initial choice made in Step 2. Children’s overreliance on the linguistic discourse is predicted to disappear with increasing processing speed.

## Discussion

In this paper, it was shown how computational modeling of referential choice within the cognitive architecture ACT-R can yield more insight into the cognitive mechanisms underlying the observed variation in speakers’ production and listeners’ comprehension of referring expressions. The ACT-R model of reference uses general memory principles of ACT-R to build a representation of the linguistic discourse, employs linguistic constraints to make an initial selection of a form or a meaning in that discourse, and performs perspective taking to check whether this initially selected form or meaning will indeed allow for mutual understanding between speaker and listener in the given discourse context. The mechanism of perspective taking does not require any additional assumptions in ACT-R. Rather, it comes for free to the model because comprehension is implemented according to the same principles of optimization as production. Perspective taking is merely modeled as the addition of an extra step of optimization from the opposite communicative perspective.

Because the ACT-R model of reference is based on verified assumptions about human cognition, the model is able to explain some of the observed variation in referential choice from variation in speakers’ and listeners’ cognitive capacities. The cognitive processes required for a specific referential task may exceed the cognitive capacities of some speakers and listeners, but not of others. Also, the cognitive processes required for some referential tasks may be more demanding than those for other tasks. Individual variation in the cognitive capacities of speakers and listeners and limitations in these capacities thus give rise to variation among and within individuals and across tasks. A first process that is expected to require sufficient cognitive capacities is the construction and maintenance of a representation of the linguistic discourse. This process is predicted by the ACT-R model to depend on the availability of sufficient WM capacity. Another process that is expected to be effortful, as it requires an additional step in production and comprehension, is perspective taking. The ACT-R model predicts that perspective taking depends on sufficient processing speed.

Hence, one source of variation in referential choice is WM capacity. Low WM capacity is argued to lead to difficulty in taking discourse prominence into account in building a representation of the linguistic discourse. Therefore, low WM capacity is expected to be involved in the production of underspecified referential forms (cf. Vogels et al., 2014). This explains why children and elderly adults occasionally produce pronouns without a clear reference in their narratives. Furthermore, an incorrect representation of the linguistic discourse due to low WM capacity is predicted to result in errors in the interpretation of pronouns as well. This explains children’s difficulty in determining the correct referent of a pronoun after a topic shift as well as adults’ child-like pattern of pronoun interpretation when their WM is taxed by an additional task.

Another source of variation in referential choice is processing speed. Insufficient speed of sentence processing is predicted to lead to a failure to consider the opposite communicative perspective. This is argued to explain children’s production of unrecoverable pronouns in narratives as well as their overly liberal interpretation of pronouns in object position. This explanation of children’s non-adult-like referential choices is in line with the view that perspective taking initially is an effortful process that requires the adjustment of one’s own perspective (e.g., Epley et al., 2004; Barr, 2008). In adults, due to the ACT-R learning mechanism of production compilation, the two-step process of perspective taking may be reduced to a one-step selection process. This could result in perspective taking processes becoming automatic and occurring early in adults (cf. Brown-Schmidt et al., 2008; Brennan and Hanna, 2009).

In recent years, several probabilistic approaches have been proposed in order to account for variation in referential choice (e.g., Frank and Goodman, 2012; van Gompel et al., 2012; Mitchell et al., 2013). For example, Frank and Goodman (2012) assume that listeners interpret referring expressions as a function of the prior probability that an object would be referred to and the probability that the speaker would use a particular word to refer to this object. In their approach, speakers are rational agents who choose words that are informative in context and reduce uncertainty about the referent. This view is criticized by Gatt et al. (2013), who argue that the observation of overspecification by human speakers provides evidence that speakers may not be rational agents after all. Like Frank and Goodman’s model, the ACT-R model of reference presented here also includes probabilistic processes, and furthermore assumes that speakers and listeners are rational agents. However, in contrast to Frank and Goodman’s model, in the ACT-R model of reference perfect rationality is not always achieved by speakers and listeners due to limitations in their cognitive capacities. Because of its bounded rationality, the ACT-R model occasionally gives rise to overspecification and underspecification.

The ACT-R model of reference is not specifically geared toward one task, but is based on general principles of human information storage, retrieval and processing in combination with general linguistic constraints on reference. Hence, the model not only explains existing data, but is also able to generate novel predictions. For example, the model predicts that speakers who are under cognitive load will produce more overly specific referring expressions after a topic shift. Also, it predicts that listeners without sufficient processing speed will be influenced in their interpretation of object pronouns by the frequencies of referents in the linguistic discourse. These predictions can be tested in new psycholinguistic experiments and in other referential tasks, providing further evidence on how referential choice varies across different tasks and among and within individuals. For example, very little is known yet about the decline of referential abilities in healthy elderly adults and how this relates to their cognitive abilities. Is it true that elderly adults’ changing performance on many cognitive tasks (including referential tasks) does not reflect cognitive decline, but instead reflects increased knowledge and corresponding memory search demands, as Ramscar et al. (2014) argue? Cognitive modeling could help to answer this question. Also, in addition to children and adults with autism or ADHD, other clinical populations could be studied that have been suggested to have limitations in their WM capacity, processing speed or both, such as patients with Alzheimer’s disease, Broca’s aphasia, or multiple sclerosis (e.g., Almor et al., 1999; Love et al., 2001; Piñango and Burkhardt, 2001). Studying these populations through cognitive modeling could reveal more about reference processing in general as well as about the cognitive deficits in these clinical populations.

The task of the ACT-R model is to make a choice between pronouns and definite descriptions in production, and between different discourse referents for a pronoun in comprehension. Most computational models for the generation of referring expressions, in contrast, focus on the choice between different definite descriptions for a particular referent, such as between *the grey desk*, *the desk facing left* and *the gray desk facing left* (Mitchell et al., 2013). To obtain a more comprehensive model of referential choice, the ACT-R model discussed here should be extended to allow for these specific referential choices between different definite descriptions as well. One proposal is by Guhe (2012), who modeled human behavior in the so-called iMAP task in ACT-R. In this task, participants had to reproduce a route on a map by referring to landmarks on the map using features such as color, number and kind (e.g., *red bugs*, *four bugs*, or *four red bugs*). Guhe developed two ACT-R models of human behavior in this task, the first one an extension of the incremental algorithm of Dale and Reiter (1995) and the second one based on a fixed template of features. Both cognitive models select features on the basis of the utility of the corresponding ACT-R production rule: production rules contributing to a successful interaction are selected with a higher probability. Furthermore, both cognitive models are able to adapt the utility value of features to feedback of whether a referring expression was used successfully. While the second model had a higher correlation with the human data, it was more geared toward the specific task. On the other hand, the first model was more general, but had difficulty predicting under- and overspecified referring expressions because of its goal to generate a uniquely distinguishing expression (for discussion, see Guhe, 2012, p. 320). However, it may be possible to circumvent this problem of the first model by replacing the goal of generating a uniquely distinguishing expression by the goal of finding a bidirectionally optimal expression, as in the ACT-R model presented here, thus aiming at avoiding misunderstanding rather than avoiding ambiguity. As the two models capture the general patterns of adaptive change in referential choice that are observed in the human data, they illustrate that modeling specific referential choices between different definite descriptions is in principle possible in ACT-R.

Another useful addition to the model would be the inclusion of visual factors in the calculation of referent activation, as the presence or absence of characters in the visual context also affects the choice between a pronoun and a full noun phrase (Fukumura et al., 2010). This would also be more in line with the view that referents are bundles of multimodal features (e.g., van der Sluis and Krahmer, 2007; van der Sluis et al., 2008), which led van der Sluis and Krahmer (2007) to include various types of pointing gestures in their computational model for the generation of referring expressions. A more realistic calculation of referent activation would also require the inclusion of further linguistic and cognitive factors, such as coherence relations between utterances, animacy, and first mention. As the first two factors have been incorporated as constraints in linguistic analyses (e.g., de Hoop, 2013; Hendriks, 2014), they may alternatively be included in the set of linguistic constraints implemented in the ACT-R model.

Despite these limitations, the ACT-R model of referential choice seems to be a promising starting point for the further exploration of factors involved in referential choice. By running computational simulations that manipulate the cognitive and linguistic factors implemented in the ACT-R model, the model can generate quantitative predictions about performance in various populations of speakers and listeners on a variety of referential tasks. These predictions can be tested experimentally, thus allowing us to gain further insights in the dependence of referential choice on these cognitive and linguistic factors.

## Author Contributions

The author confirms being the sole contributor of this work and approved it for publication.

## Conflict of Interest Statement

The author declares that the research was conducted in the absence of any commercial or financial relationships that could be construed as a potential conflict of interest.
